# Survival from cancer of the larynx in England and Wales up to 2001

**DOI:** 10.1038/sj.bjc.6604581

**Published:** 2008-09-23

**Authors:** B Rachet, M J Quinn, N Cooper, M P Coleman

**Affiliations:** 1 Cancer Research UK Cancer Survival Group, Non-Communicable Disease Epidemiology Unit, Department of Epidemiology and Population Health, London School of Hygiene and Tropical Medicine, Keppel Street, London WC1E 7HT, UK; 2 Social and Health Analysis and Reporting Division, Office for National Statistics (Room FG/114), 1 Myddelton Street, London EC1R 1UW, UK

Cancer of the larynx is one of the more common malignancies in England and Wales (ranking 20th in both sexes combined). Approximately 1800 new cases are diagnosed each year, 80% of them in men. Incidence rates are approximately 6.2 and 1.3 per 100 000 per year in men and women, respectively. Incidence has fallen by approximately 5% in men over the last decade, but little change has occurred in women. Laryngeal cancer is rare under the age of 40, but the risk rises rapidly with age. There is a marked socioeconomic gradient, with risk twice as high in the most deprived groups as in the most affluent groups (Quinn *et al*, 2001). Geographic variation in risk is also wide, with incidence less than 70% of the United Kingdom and Ireland average in southwest England and parts of the southeast, but 50% or more above the average in much of Scotland and in the main urban areas of northwest and northeast England. The combined effect is a striking regional disparity in the socioeconomic profile of the disease. In the Oxford region, for example, 50% of cases occur in affluent groups, although in the West Midlands and the northwest, that proportion is approximately 20%, with 65% of cases among the most deprived (data not shown). The annual death rate of laryngeal cancer in England and Wales is approximately 2.3 per 100 000 in men (570 deaths a year) and 0.6 in women (150 deaths a year).

The main risk factors for laryngeal cancer are alcohol and tobacco, and their effects are synergistic (Tuyns and Audigier, 1976; Tuyns *et al*, 1988). Tobacco dominates the risk for cancers of the vocal cords and glottis, whereas alcohol is more prominent for cancers of the supraglottis. This has a direct impact on survival in men and women for all laryngeal cancers combined, because the main causal exposures and the most common anatomic location of tumours within the larynx differ between the sexes, as do their diagnosis, treatment and outcome. Glottal cancers are more common in men; they give rise to hoarseness when the tumour is still small. They can often be treated surgically and are responsive to radiotherapy. They tend to have higher survival than supraglottic tumours. Cancers of the supraglottis are more common in women and do not give rise to early symptoms of hoarseness. Diagnosis from dysphagia or sore throat is often later than for cancers of the glottis, curative radiotherapy and surgery may be less successful, and survival is lower.

Survival analyses are reported here only for men. Some 20 000 men were diagnosed with a first, primary, invasive malignancy of the larynx in England and Wales during the period 1986–1999, and followed up to the end of 2001, approximately 89% of those eligible for analysis. Approximately 2% were excluded because their vital status was unknown on 5 November 2002, when the data were extracted for analysis; 4% because the laryngeal cancer was not their first primary cancer and another 4% because their survival was zero or unknown, most of whom were registered from a death certificate only.

Half (49%) of the laryngeal tumours diagnosed in men during the 1990s arose in the glottis (endolarynx), including the vocal cords. The increase of approximately 5% since the 1980s is matched by a similar drop in the proportion of tumours of unspecified subsite (down to 31%), suggesting gradual improvement in diagnostic precision. Approximately 16% arose in the supraglottis (epilarynx). Tumours of the larynx below the cords (subglottis) remained rare (1.3%). Almost 85% of laryngeal tumours diagnosed during the 1990s were squamous carcinomas, an increase of 6% since the 1980s, matched by a similar fall in the proportion coded as carcinoma without further specification (down to 7%), again suggesting improved precision of pathology. Verrucous carcinoma was specified as often in the 1990s alone (125 cases, 1%) as in the earlier two decades combined (130 cases, 0.4%), but adenocarcinoma remains rare (0.3%).

## Survival trends

**Figure 1:**
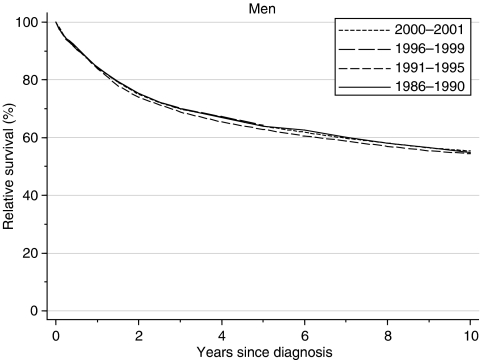
Relative survival (%) up to 10 years after diagnosis by calendar period of diagnosis: England and Wales, adults (15–99 years) diagnosed during 1986–1999 and followed up to 2001. Survival estimated with cohort or complete approach (1986–1990, 1991–1995, 1996–1999) or hybrid approach (2000–2001) (see Rachet *et al*, 2008).

Relative survival from laryngeal cancer in men diagnosed during the 1990s was only slightly higher than for men diagnosed during the late 1980s, at approximately 84, 64 and 54% at 1, 5 and 10 years, respectively (Table 1 and Figure 1). After adjustment for deprivation, however, the estimate of trend in 5-year survival was an increase of 3.3% every 5 years between 1986–1990 and 1996–1999, a trend of borderline significance (95% confidence interval 0.0–6.7%). This rate of increase in survival is adjusted for the deprivation gap in survival and for any changes in the distribution of patients by deprivation category, and it is a more reliable estimate of the trend in survival than would appear from the very similar survival of 63–64% in successive calendar periods.

Predicted survival derived from the hybrid approach (Brenner and Rachet, 2004) using survival probabilities observed during 2000–2001 does not suggest any imminent increase in survival.

## Deprivation

**Figure 2:**
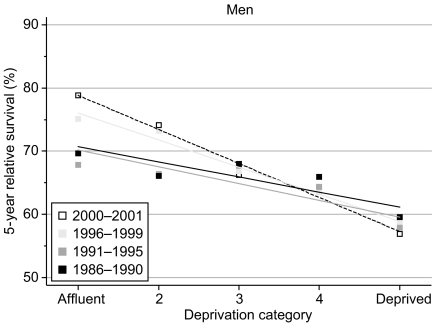
Trends in the deprivation gap in 5-year relative survival (%) by calendar period of diagnosis: England and Wales, adults (15–99 years) diagnosed during 1986–1999 and followed up to 2001.

Five-year survival was 17% lower (95% confidence interval 12–22% lower) among men diagnosed in the most deprived group in 1996–1999 than those in the most affluent group (Table 2, Figure 2). This is the steepest socioeconomic gradient in survival among all 20 common cancers that we examined, and it has widened more rapidly – by 3.7% every 5 years – than for any other cancer in men, even prostate cancer (q.v.). Figure 2 shows that virtually all the overall increase in 5-year survival between 1986–1990 and 1996–1999 occurred among the more affluent groups, while it stagnated or even fell slightly amongst men in the more deprived groups.

The deprivation gap in 10-year survival for men diagnosed during the early 1990s was also very wide, at 11%.

Short-term prediction of the deprivation gap in 5- and 10-year survival between the most affluent and most deprived groups suggests that the socioeconomic disparity in survival may widen still further, to 20% or more, in the near future (Table 2).

## Comment

Survival from laryngeal cancer in men in England and Wales did not increase rapidly in the 15 years to the end of the 20th century, and such increases as did occur were virtually confined to men in the most affluent sectors of society. The disparity in survival between rich and poor is now the widest of any common cancer, and it has worsened more rapidly than for any other cancer in men. The deprivation-specific survival estimates take account of socioeconomic differences in background mortality and trends in those differences over time, just as in the analyses for other cancers, so this unusually large increase in the inequality of cancer survival demands an explanation.

**Figure 3:**
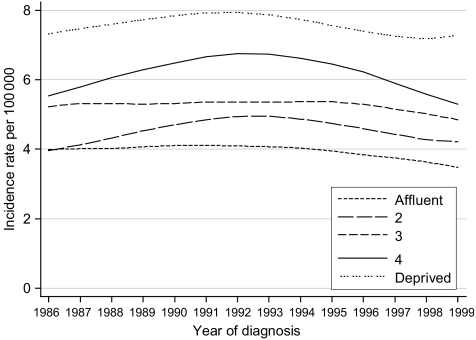
Trends in the age-standardised incidence of laryngeal cancer in men aged 15–99 years, by deprivation group: England and Wales, 1986–99.

Incidence trends in all socioeconomic groups were broadly parallel, showing a gentle and symmetrical increase, plateau and decline over the 14-year period 1986–1999 (Figure 3). The incidence trends do not suggest an artefact of diagnosis or registration that might account for the different survival trends between socioeconomic groups.

The vast majority of laryngeal cancers are related to alcohol and or tobacco, so the underlying risk of death in these patients from any cause of death related to tobacco or alcohol is probably even higher than that of men in the same socioeconomic group in the general population. Even life tables that are specific to each socioeconomic group may therefore still under-estimate the true background mortality of these men to some extent. Relative survival estimates on the basis of such life tables, although they are in principle adjusted for mortality not related to laryngeal cancer, may thus underestimate the cancer-specific survival of men with laryngeal cancer to some degree.

This cannot be the only explanation, however, as the same life tables were used in the survival analyses for all cancers, and the deprivation gap in survival for other cancers for which tobacco or alcohol are causal factors (oesophagus, pancreas, kidney and bladder) was stable, or did not increase nearly as much as it did for laryngeal cancer. Further, the deprivation gap in 5-year survival for laryngeal cancer seen in men diagnosed during 1986–1990 (approximately −10%) had been fairly stable since the 1970s (Coleman *et al*, 1999), so deaths from other tobacco-related causes cannot readily explain the increase in the deprivation gradient for relative survival from laryngeal cancer during the 1990s. Life tables that are specific to such a group are not available, but survival estimates made with approximate life tables for smokers, derived from a cohort study (Cutler and Ederer, 1958), do not suggest this could account for much of the difference in relative survival.

The increasing difference in survival between socioeconomic groups could thus reflect a deprivation gradient in the quality of care for diseases related to alcohol and tobacco.

## Figures and Tables

**Table 1:** Trends in relative survival (%) by time since diagnosis and calendar period of diagnosis: England and Wales, adults (15–99 years) diagnosed during 1986–1999 and followed up to 2001

		**Calendar period of diagnosis^a^**									
		**1986–1990**		**1991–1995**		**1996–1999**		**Average change (%) every 5 years^b^**		**Prediction^c^ for patients diagnosed during 2000–2001**	
**Time since diagnosis**		**Survival (%)**	**95% CI**	**Survival (%)**	**95% CI**	**Survival (%)**	**95% CI**	**Survival (%)**	**95% CI**	**Survival (%)**	**95% CI**
1 year	Men	**84.3**	(83.3, 85.2)	**83.9**	(82.9, 84.8)	**84.4**	(83.3, 85.4)	**1.2**	(−0.9, 3.2)	**84.2**	(82.6, 85.6)
5 years	Men	**63.9**	(62.5, 65.3)	**62.9**	(61.5, 64.2)	**64.3**	(62.5, 66.1)	3.3*	(0.0, 6.7)	**63.9**	(61.7, 66.1)
10 years	Men	**54.9**	(53.2, 56.5)	**54.4**	(52.5, 56.2)			**−0.8**	(−8.1, 6.5)	**55.4**	(52.7, 57.9)

CI=confidence interval.

a Survival estimated with cohort or complete approach (see Rachet *et al*, 2008).

b Mean absolute change (%) in survival every 5 years, adjusted for deprivation (see Rachet *et al*, 2008).

c Survival estimated with hybrid approach (see Rachet *et al*, 2008).

^*^P<0.05.

**Table 2:** Trends in the deprivation gap in relative survival (%) by time since diagnosis and calendar period of diagnosis: England and Wales, adults (15–99 years) diagnosed 1986–1999 and followed up to 2001

		**Calendar period of diagnosis^a^**									
		**1986–1990**		**1991–1995**		**1996–1999**		**Average change (%) every 5 years^b^**		**Prediction^c^ for patients diagnosed during 2000–2001**	
**Time since diagnosis**		**Deprivation gap (%)**	**95% CI**	**Deprivation gap (%)**	**95% CI**	**Deprivation gap (%)**	**95% CI**	**Deprivation gap (%)**	**95% CI**	**Deprivation gap (%)**	**95% CI**
1 year	Men	−5.3**	(−8.1, −2.6)	−5.6**	(−8.4, −2.9)	−7.7**	(−10.8, −4.7)	**−1.2**	(−3.4, 0.9)	−9.6**	(−13.9, −5.2)
5 years	Men	−9.6**	(−13.7, −5.4)	−10.6**	(−14.6, −6.6)	−17.2**	(−22.4, −11.9)	−3.7*	(−7.1, −0.2)	−21.5**	(−28.0, −15.1)
10 years	Men	−11.2**	(−16.2, −6.3)	−11.1**	(−16.8, −5.5)			**0.1**	(−7.4, 7.6)	−23.4**	(−31.3, −15.6)

CI=confidence interval.

a Survival estimated with cohort or complete approach (see Rachet *et al*, 2008).

b Mean absolute change (%) in the deprivation gap in survival every 5 years, adjusted for the underlying trend in survival (see Rachet *et al*, 2008).

c Survival estimated with hybrid approach (see Rachet *et al*, 2008).

^*^P<0.05; ^**^P<0.01.
